# Peripheral Analgesic Effect of a Novel Curcuminoid Derivative: Possible Involvement of Peripheral Opioid Receptor and ATP-Sensitive Potassium Ion Channel

**DOI:** 10.3390/pharmaceutics18010141

**Published:** 2026-01-22

**Authors:** Ming Tatt Lee, Yu-Cheng Ho, Chau Ling Tham, Ahmad Akira, Nordin Lajis, Daud Ahmad Israf, Mohd Roslan Sulaiman

**Affiliations:** 1 Office of Postgraduate Studies, UCSI University, Kuala Lumpur 56000, Malaysia; 2 College of Health Sciences, Chang-Jung Christian University, Tainan 711301, Taiwan; 3 School of Medicine, College of Medicine, I-Shou University, Kaohsiung 84001, Taiwan; 4 Department of Biomedical Science, Faculty of Medicine and Health Sciences, Universiti Putra Malaysia, Serdang 43400, Malaysia; 5 Laboratory of Natural Products, Institute of Bioscience, University Putra Malaysia, Serdang 43400, Malaysia

**Keywords:** peripheral analgesia, inflammatory mediators, opioid receptor, cyclic guanosyl monophosphate, potassium ion channels, good health and well-being

## Abstract

**Background/Objectives:** The present study investigated the local analgesic effect of a novel synthetic cyclohexanone derivative, 2,6-bis-4-(hydroxyl-3-methoxybenzilidine)-cyclohexanone, or BHMC, in a mouse model of peripheral nociception. **Methods:** Local administration of BHMC (0.5–60 µg/paw) intra-plantarly in the hindpaws of mice exhibited significant inhibition in carrageenan-induced paw hyperalgesia. Intra-plantar pretreatment of naloxone (non-selective opioid receptor blocker), D-Phe-Cys-Tyr-D-Trp-Orn-Thr-Pen-ThrNH2 (CTOP, selective µ-opioid receptor blocker), and nor-binaltorphimine (nor-BNI, selective κ-opioid receptor blocker), but not naltrindole hydrochloride (selective δ-opioid receptor blocker), reversed the anti-nociceptive effect of BHMC. The peripheral analgesic effect of BHMC was also reversed by intra-plantar pretreatment of methylene blue (soluble guanosyl cyclase blocker), but not N^G^-nitro-L-arginine (L-NAME, nitric oxide synthase blocker). Involvement of the potassium channel in the local analgesic effect of BHMC was shown through the reversed analgesic effect by intra-plantar pretreatment of glibenclamide (ATP-sensitive potassium channel blocker), but not by charybdotoxin (large-conductance calcium-sensitive potassium channel blocker), apamin (small-conductance calcium-sensitive potassium ion channel blocker), or tetraethylammonium (voltage-sensitive potassium channel blocker). **Results:** Taken together, the present study demonstrated that the local administration of BHMC attenuated nociception, with possible mechanisms that may involve the desensitization of inflammatory mediators’ receptors, opioid receptor activation, and nitric oxide-independent cyclic guanosine monophosphate activation of ATP-sensitive potassium ion channel opening. **Conclusions:** The current findings may further support the exploration of BHMC as a new therapeutic agent for pain and inflammation, for the betterment of human health.

## 1. Introduction

Pain remains as one of the main unmet medical needs. The current pharmacotherapy for pain, although effective, is hindered by the potential adverse effects of long-term use [1]. In some cases, patients with chronic pain demonstrated a refractory response to current medications. Chronic pain is known to be one of the underlying causes for various neuropsychiatric conditions [2,3]. These phenomena warrant the exploration of novel analgesic agents.

Cyclohexanone, a six-membered ring ketone, and its derivatives are reported to possess numerous applications in pharmaceutical and agrochemical industries [4,5]. Benzilidines, particularly of cyclo-conjugated bis(benzilidine)ketone families of compounds, have undergone intensive research due to their potential therapeutic effects on the biological system. Bis(benzilidine) ketones exhibit considerable cytotoxicity in preclinical cancer models [6,7,8,9]. Moreover, benzilidine cyclohexanone derivatives have been popularized in the synthetic pharmacological community since the 1980s [10]. Patents had been granted to a research group from the University of Gadjah Mada, Yogyakarta, Indonesia, for the formulation, synthesis, and proven biological effects of various benzilidine cyclohexanone derivatives [11,12]. These biological effects include anti-inflammation, anti-oxidation, anti-bacterial, and anti-fungal properties.

**Figure 1:**
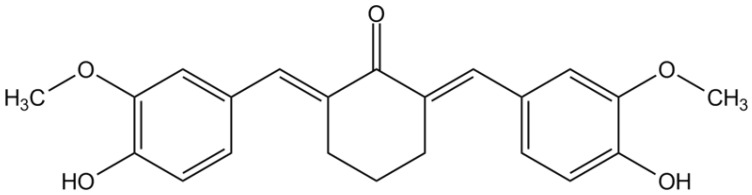
Chemical structure of 2,6-bis-4-(hydroxyl-3methoxybenzilidine)-cyclohexanone (BHMC).

Our research group has previously introduced a synthetic curcuminoid derivative, namely 2,6-bis-4-(hydroxyl-3-methoxybenzilidine)-cyclohexanone or BHMC (Figure 1). This curcuminoid derivative showed potential anti-inflammatory [13,14] and anti-cancer effects [15,16] in vitro. Moreover, BHMC exhibited significant antipruritic [17], anti-asthmatic [18], and analgesic [19,20] effects in vivo through systemic administration. Interestingly, we also demonstrated that BHMC at similar doses is capable of suppressing chronic pain in the form of neuropathic pain [21]. However, the main target for systemic analgesic effect of BHMC was reported to mediate through opioid receptors, as it was shown to be reversible by naloxone pretreatment [19,21], a non-selective opioid receptor antagonist. Given that systemic and central-acting opioid agonists are known to induce unwanted psychomotor effects, e.g., tolerance, dependence, euphoria, etc. [22,23], this poses a challenge in developing novel analgesic agents with opioidergic affinity.

Besides the central nervous system, opioid receptors are also expressed in the peripheral nervous system, especially the nerve endings in the nociceptors [24,25]. Thus, the local administration of opioid activator would be a viable strategy to bypass or avoid the unwanted central effects of opioid activation [26]. The previous literature has demonstrated the potential and effectiveness of targeting peripheral/local opioid receptors in pain modulation [26,27]. In the present study, we investigated the peripheral analgesic effect of BHMC, with possible inactivation of nociceptive protein kinases and the opening of potassium channels related to opioid receptor activation.

## 2. Materials and Methods

### 2.1. Animals

Male Balb/C mice of 8 weeks old (20–25 g) were housed under the conditions of 25–30 °C and a 12 h light–dark cycle. Mice were acclimatized for at least 1 week before beginning each experiment and received water and food ad libitum. Experiments reported in this study were performed in accordance with ARRIVE guidelines, approved by the Ethical Committee, Faculty of Medicine and Health Sciences, Universiti Putra Malaysia (ACUC_UPM/FPSK/PADS/BR-UUH/00330). The animals from each cohort were randomly allocated to different treatment groups via random sorting. Animals that showed signs of injury due to in-cage fighting would be excluded. However, no animals were excluded from this study. The number of animals and the intensity of noxious stimuli used were minimal and just necessary to demonstrate the consistent effects of the drug treatments. Each animal was only exposed to one experiment and was euthanized via cervical dislocation at the end of the study.

### 2.2. Measurement of Hyperalgesia

An amount of 20 µL of carrageenan suspension (2% *w*/*v*) was injected subcutaneously into the intra-plantar region to induce hyperalgesia in the hindpaws. The degree of hyperalgesia was measured according to the mechanical paw pressure test described by Randall and Selitto [28]. An analgesiometer (IITC, San Francisco, CA, USA), equipped with a cone-shaped paw-presser with a rounded tip, was applied with gradually increasing force to the dorsal surface of the hindpaw. The pressure in weight (g) required to elicit the withdrawal of the inflamed paw was determined as the nociceptive threshold. A cut-off value of 220 g was used to prevent excessive damage to the animal. The nociceptive threshold was measured before carrageenan injection and 3 h post carrageenan injection. The results were calculated by the difference between the pre-induction threshold and the post-induction threshold.

### 2.3. Experimental Protocol

The inflamed paws were measured with Randall Selitto analgesiometer 3 h post carrageenan injection to obtain nociceptive threshold level [29]. To evaluate the local analgesic effect of BHMC, BHMC (0.5, 1.0, 5.0, 10, 20, 40, and 60 µg/paw) was intra-plantarly (i.pl.) administered into the left hindpaw 15 min before paw pressure measurement. To exclude the possibility of interference from the systemic analgesic effect of BHMC, carrageenan was injected into the right hindpaw of the same animal without it being given BHMC treatment, and the nociceptive threshold of the right hindpaw was measured as well. For the subsequent mechanism of action study, the dosage of 20 µg/paw of BHMC was selected for further study. Antagonists of opioid receptor subtypes, nitric oxide (NO) synthase, guanylate cyclase, and K^+^ channel subtypes were administered 5 min prior to BHMC treatment in the carrageenan-induced hyperalgesia protocol mentioned above to study the involvement of opioid receptor/NO/cGMP/K^+^ channels in the peripheral analgesic effect of BHMC. The protocols above were assessed in pilot studies to determine the most suitable time points of injection and the optimum dose of each substance to have the desired effects based on the previous literature [29,30,31].

### 2.4. Chemicals

All hyperalgesia-inducing agents and antagonists were purchased from Sigma Aldrich, St. Louis, MO, USA. BHMC or 2,6-bis-4-(hydroxyl-3-methoxy-benzilidine)-cyclohexanone was chemically synthesized at the Institute of Bioscience, Universiti Putra Malaysia, as per previous published protocols [14,19,32]. The structure of BHMC was determined by nuclear magnetic resonance and mass spectrometry. BHMC is 99.9% pure as determined by high-performance liquid chromatography. BHMC was dissolved in a vehicle comprised of 5% absolute ethanol, 5% Tween 20, and 90% distilled water before being administered into the animal.

### 2.5. Statistical Analysis

The data are expressed as the mean ± S.E.M. One-way analysis of variance (ANOVA) was used to compare the effect of BHMC with the negative control group, as well as compare with the antagonist co-administered groups, followed by Sidak’s post hoc test. Results with *p* < 0.05 were considered statistically significant.

## 3. Results

### 3.1. Peripheral Analgesic Effect of BHMC

**Figure 2:**
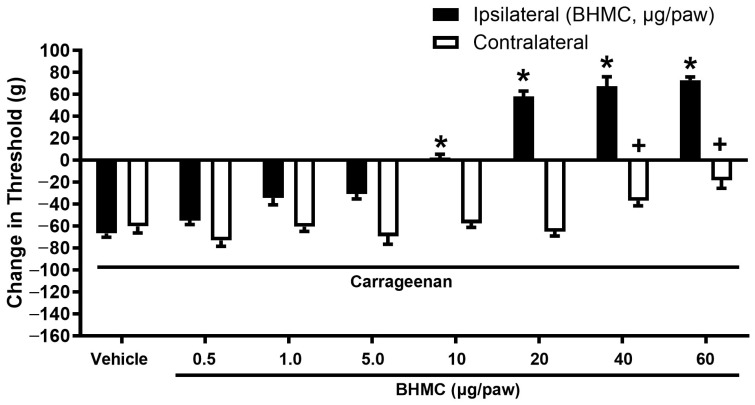
Effect of local administration of BHMC (0.5–60 µg/paw, i.pl.) on ipsilateral and contralateral nociceptive threshold change induced by carrageenan injection. Each mouse was subjected to intra-plantar (i.pl.) injection of carrageenan on both paws. The nociceptive threshold was measured by the analgesiometer 3 h post-carrageenan injection. BHMC (i.pl.) was administered to the left hindpaw 15 min before the nociceptive measurement. Each column represents mean ± S.E.M with *n* = 8. * denotes *p* < 0.05, significant difference between nociceptive threshold change in the ipsilateral BHMC and vehicle-treated paw. + denotes *p* < 0.05, significant difference between nociceptive change in contralateral BHMC and vehicle-treated paw.

In the carrageenan-induced hyperalgesia test, local administration of BHMC (0.5–10 µg/paw) showed a significant anti-hyperalgesia effect on the ipsilateral paw of each mouse. In higher doses of BHMC (20–60 µg/paw), the results showed that the pain threshold of treated mice towards mechanical nociception was elevated compared to a pre-carrageenan-induction level (Figure 2). It is noteworthy that BHMC at 20 µg/paw exerted an anti-hyperalgesia effect and caused an elevated pain threshold without causing an anti-nociceptive effect on the contralateral paw, whereas a higher amount of BHMC would have a systemic anti-nociceptive effect.

### 3.2. Involvement of Opioid Receptors in the Peripheral Analgesic Effect of BHMC

**Figure 3:**
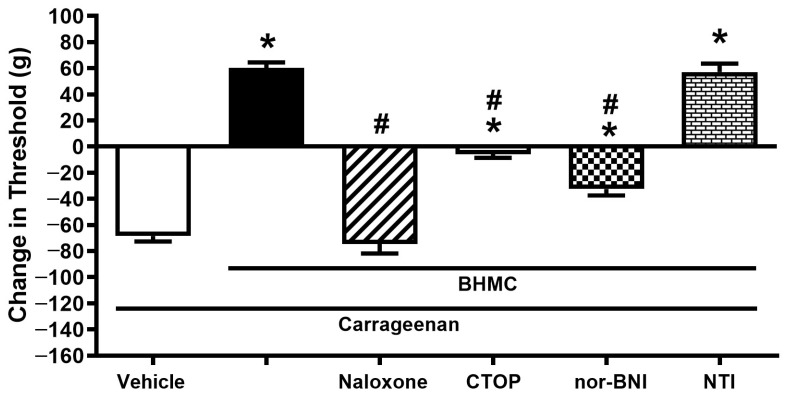
Effect of the local pretreatment of various opioid receptor antagonists on the peripheral analgesic effect of BHMC in the carrageenan-induced hyperalgesia test. The experimental timeline is similar to Figure 2, with the antagonists being administered i.pl. to the left hindpaw 5 min before BHMC injection. Each column represents mean ± S.E.M with *n* = 8. * denotes *p* < 0.05, significant difference in nociceptive threshold change between vehicle-treated paw and BHMC+antagonist-treated paw. # denotes *p* < 0.05, significant difference in nociceptive threshold change between BHMC-treated groups and BHMC+antagonist-treated groups.

Pretreatment of naloxone (100 µg/paw, i.pl.) reversed the peripheral analgesic effect of BHMC (20 µg/paw, i.pl.). Further investigation on opioid subtypes showed that pretreatment with CTOP (10 µg/paw, i.pl.) and nor-BNI (40 µg/paw, i.pl.) significantly reversed the anti-hyperalgesia effect of BHMC, whereas pretreatment with NTI (20 µg/paw, i.pl.) did not affect the effect of BHMC (Figure 3).

### 3.3. Involvement of NO-cGMP in Peripheral Analgesic Effect of BHMC

**Figure 4:**
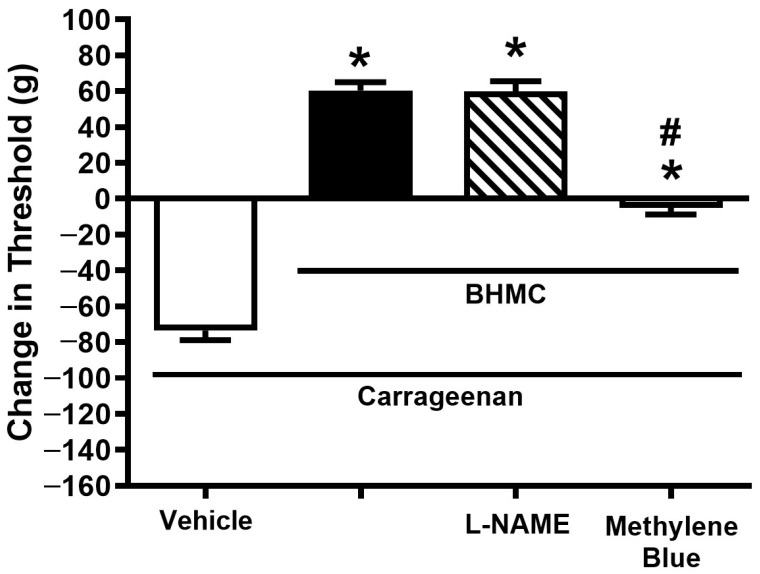
Effect of local pretreatment of NOS inhibitor (L-NAME) and guanylate cyclase inhibitor (methylene blue) on the local analgesic effect of BHMC (20 µg/paw, i.pl.) in the carrageenan-induced hyperalgesia test. The experimental timeline is similar to Figure 3. Each column represents the mean ± S.E.M with *n* = 8. * denotes *p* < 0.05, significant difference in nociceptive threshold change between vehicle-treated paw and BHMC+antagonist-treated paw. # denotes *p* < 0.05, significant difference in nociceptive threshold change between BHMC-treated groups and BHMC+antagonist-treated groups.

Pre-treatment with NO synthase inhibitor, L-NAME (20 µg/paw, i.pl.) did not cause any effect on the peripheral analgesic effect of BHMC, whereas the modulation of the anti-nociceptive effect of BHMC was observed in the pre-administered group with methylene blue (20 µg/paw, i.pl.), a guanylate cyclase inhibitor (Figure 4).

### 3.4. Involvement of K^+^ Channels in Peripheral Analgesic Effect of BHMC

**Figure 5:**
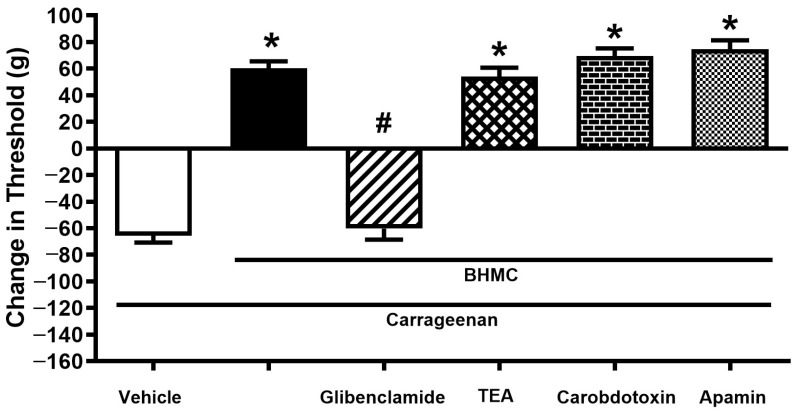
Effect of local pretreatment of various K^+^ channel antagonists on the local analgesic effect of BHMC (20 µg/paw, i.pl.) in the carrageenan-induced hyperalgesia test. The experimental timeline is similar to Figure 3 and Figure 4. Each column represents the mean ± S.E.M with *n* = 8. * denotes *p* < 0.05, significant difference in nociceptive threshold change between vehicle-treated paw and BHMC+antagonist-treated paw. # denotes *p* < 0.05, significant difference in nociceptive threshold change between BHMC-treated groups and BHMC+antagonist-treated groups.

Glibenclamide (40 µg/paw, i.pl.) antagonized the peripheral analgesic effect of BHMC (20 µg/paw, i.pl.). In contrast, apamin (0.2 µg/paw, i.pl.), charybdotoxin (0.4 µg/paw, i.pl.), and tetraethylammonium (20 µg/paw, i.pl.) did not show significant attenuation on the anti-hyperalgesic effect of BHMC (Figure 5).

## 4. Discussion

Throughout the years, pharmacologists have relied on analogue compounds to search for potential novel therapeutic substances. Aspirin, indomethacin, dexamethasone, etc., are among examples of derivative-based analgesics. A synthetic benzilidine-cyclohexanone analogue, in our previous study, modulated the nociceptive reaction in mice through systemic administration [19,21]. The present study demonstrated the local analgesic effect of a synthetic benzilidine cyclohexanone derivative, 2,6-bis-4-(hydroxyl-3methoxybenzilidine)-cyclohexanone, or BHMC, and its possible mechanisms of action.

In the present study, the carrageenan-induced inflammatory pain model in mice was used as the test model. This model is known to consistently induce pain and inflammatory responses in rodents when administered via the i.pl. route. Acute and localized pain responses in this model are known to be mediated by biphasic release of inflammatory mediators such as histamine, prostaglandins, and cytokines, which activate the nociceptive receptors. In the experimental group which animals received a lower amount of BHMC (0.5, 1.0, 5.0, and 10 µg/paw), significant reversal of pain reaction was observed, and 10 µg/paw of BHMC injection restored the pain threshold of the inflamed paw to pre-carrageenan injection levels; whereas, in groups that received a higher amount of BHMC (20, 40, and 60 µg/paw), the pain threshold further elevated, implying that BHMC may be able to grant the injured paw “extra-resistance” towards excessive mechanical force. We suspected that a high dose of BHMC would possibly desensitize the mechanical nociceptors of the treated region of the paw. Nonetheless, it is important to take note that the present study is unable to rule out the possibility of the contribution of local motor impairment or local sensory suppression for this observation, which warrants further exploratory study in the future. To investigate the mechanism of action of the peripheral analgesic effect of BHMC, 20 µg/paw of BHMC was selected, as it provided the maximum local analgesic effect against carrageenan-induced hyperalgesia and local mechanical nociception, ipsilaterally.

During the event of inflammation or tissue injury, chemical mediators and excitatory amino acids were released, increasing the sensitivity of nociceptors on primary afferent neurons, thus transducing innocuous stimuli (thermal or mechanical) as pain, a phenomenon named hyperalgesia. Substance P [33], bradykinin [34], and prostaglandin E_2_ [35] are the key mediators, along with glutamate [36], an excitatory amino acid, released after carrageenan injection. They are responsible for the sensitization and activation of nociceptors of primary afferent neurons. Primary afferent sensitization is a metabotropic phenomenon that results in lowering the nociceptor threshold, which involves stimulating G protein-coupled receptors by these inflammatory mediators [37]. Protein kinase A (PKA) is activated through adenylyl cyclase/cAMP/PKA second messenger cascade via PGE_2_ receptor activation [38], whereas protein kinase C (PKC) is activated through PIP_2_-PLC-DAG-PKC cascade upon bradykinin B2 receptor activation [39]. Ultimately, K^+^ channels are shut [40], causing depolarization of primary afferent neurons. In the present study, local administration of BHMC alleviated hyperalgesia caused by carrageenan injection in mice, possibly through the desensitization of nociceptors by BHMC and/or the inhibition of nociception-related protein kinase formations/functions. However, further studies on the involvement of tetrodotoxin-resistant Na^+^ channels [41], N-, and P/Q type Ca^2+^ channels [42] should be carried out, as their involvement is prominent in upregulating pain responses during inflammation.

In our previous study, systemic administration of naloxone reversed the analgesic effect of systemically injected BHMC, thus showing the involvement of the opioidergic system in the analgesic effect of BHMC. In the present study, we hypothesize that peripheral opioid receptors are involved in the peripheral analgesic effect of BHMC. Opioid receptors are expressed in peripheral afferent neurons; the analgesic effects of opioid drugs can be mediated by peripheral opioid receptors located on these neurons [43]. It is evident that µ-, κ-, and δ-subtypes of opioid receptors are involved in peripheral analgesia. Stein et al. [44] demonstrated that local injection of ipsilaterally active doses of µ-, κ-, and δ-opioid receptor agonists in the inflamed paw produced significant analgesia, which was reversible by selective antagonists. Findings from the present study showed that pre-treatment with µ- and κ-opioid receptor antagonists, but not δ-opioid receptor antagonists, reversed the peripheral analgesic effect of BHMC. This implied that µ- and κ-opioid receptors are for the peripheral anti-hyperalgesia activity of BHMC, although a number of studies have demonstrated examples of opioid drugs acting via µ-agonist/κ-antagonist (or vice versa) properties, such as nalbuphine [45]. However, an exception to this “characteristic” was shown in a study demonstrating that norbuprenorphine [46] exhibited partial agonistic activity on both µ and κ receptors. Further investigation on full or partial agonism of BHMC towards µ- and κ-opioid receptors in primary afferent neurons is required to further establish the pharmacological profile of BHMC as a peripheral analgesic agent.

Previous studies showed that all three subtypes of opioid receptor agonists produce analgesic effects through the NO-cGMP pathway [47,48,49]. In the present study, the involvement of NO in the analgesic effect of BHMC had been ruled out since pretreatment with L-NAME, a nitric oxide synthase inhibitor, failed to reverse the peripheral analgesic effect of BHMC. However, the possible involvement of cGMP is observed, as pretreatment with the guanylate cyclase inhibitor, methylene blue, reversed the inhibitory effect of BHMC in inflammatory pain. The present results indicated that BHMC did not mediate its anti-nociceptive action in opioid-NO-cGMP-K^+^ channel pathways as compared to other opioid drugs in the literature [50], with the absence of the participation of an NO molecule. In this context, we suspect that BHMC interacts with guanylate cyclase independent of an NO molecule, thus producing an analgesic effect. There are a number of studies reporting on NO-independent activation of cGMP by opioid agonists. Duarte and Ferreira [51] suggested that activation of cGMP, which takes part in the central action of morphine (µ- receptor agonist), was via other sources and not the L-arginine–NO pathway, as an NO synthase inhibitor failed to reverse the analgesic effect of morphine. However, Ferreira et al. (1991) earlier demonstrated that L-Arginine-cGMP underlined the peripheral analgesic effect of morphine [47]. These observations suggest that NO oxide dependency on opioid analgesia may vary between sites of action and may vary between different opioid drugs. Gultekin et al., 2006, demonstrated that venlafaxine, a serotonin reuptake inhibitor that possesses κ and δ opioid analgesic effects [52], exhibited opioid agonistic activity with no relationship with NO activation [53]. Though it is common in the literature that the guanylate cyclase is activated by an NO molecule to produce cGMP for analgesic purposes, NO-independent activation of guanylate cyclase is available in the literature. Peripheral administration of atrial natriuretic peptide produces a local analgesic effect by increasing cGMP content and K^+^ channel activation in a NO-independent manner [54], a mechanism similar to its vasodilating effect [55,56,57]. In a study performed by Pei et al., 2003, it was found that κ-opioid receptor stimulation contributes to aortic artery dilation through the activation of ATP-sensitive K^+^ channels in rats, without the involvement of NO molecules [58]. Although this publication concerns different systemic pathways of κ receptors, it poses the possibility that κ receptor agonists may activate ATP-sensitive K^+^ channels through NO-independent pathways, coinciding with the similar mechanism of action of atrial natriuretic peptide in both the neuronal and cardiovascular systems, as discussed earlier. Further investigation into in-depth molecular pathways ought to be carried out to investigate the mechanisms by which BHMC deviates from other opioid drugs in the NO/cGMP pathway.

It is noteworthy that the local inhibitory effect of BHMC on carrageenan-induced paw hyperalgesia can also be attributed to opioid receptor activation, where the inhibition of adenyl cyclase is accomplished by binding the Gα subunit, subsequently decreasing cAMP and PKA production [43]. Further study on the involvement of Na^+^ and Ca^2+^ channels should be carried out, as mentioned earlier, as they are regulated by the Gβγ subunit of the Gi/o protein complex of opioid receptors [59]. Another speculation is that protein kinase G (PKG), a constituent of the L-Arginine/NO/cGMP/PKG/K^+^ channel pathway, is involved in the anti-nociception activity, which might lead to a decreased influx of Na and Ca ions and/or increased hyperpolarization by opening the K^+^ channel [60].

The antagonism of BHMC in the ATP-sensitive K^+^ channel is observed in the present study. Pretreatment with glibenclamide, a sulphonylurea subtype, which is known for its selective antagonism towards ATP-sensitive K^+^ channels, reversed the peripheral analgesic effect of BHMC, suggesting that hyperpolarization of excitable neurons led by ATP-sensitive K^+^ channel opening might be a key factor in primary afferent neuron desensitization. On the contrary, apamin (a selective blocker of small conductance Ca^2+^-activated K^+^ channels), charybdotoxin (a selective blocker of large conductance Ca^2+^-activated K^+^ channels), and TEA (a voltage-gated K^+^ channel blocker) did not reverse the peripheral analgesic effect of BHMC. These findings implied that BHMC may, either through direct binding or indirect chemical reactions, selectively activate ATP-sensitive K^+^ channel opening. The association of K^+^ channel opening with cGMP increase in primary afferent neurons was proven by Sachs et al. (2003) to be mediated through L-arginine/NO production by nitric oxide synthase [60]. Although the present results implied that the increase in cGMP by BHMC is possibly activated by sources other than NO, the consequence of this is eventually related to the opening of ATP-sensitive K^+^ channels to hyperpolarization of primary afferent neurons. Furthermore, subsequent up-lifting of the intracellular level of cGMP, resulting in the opening of ATP-sensitive K^+^ channels, is proven to be a consequence of guanylate cyclase activation [61].

Nonetheless, one of the limitations of the current study is the lack of molecular verification of the interaction of BHMC with the postulated pathways. An electrophysiological study involving the measurement of neuronal currents would be one of the strategies to directly observe the response of nociceptive neurons with BHMC and the abovementioned signaling antagonists. These observations would be significant in discerning the actual mechanism of action of BHMC at molecular and/or receptor levels. Furthermore, genetically modified animal models such as µ- and/or κ-opioid receptor-knockout mice would be a useful model to discern if BHMC can exert its peripheral analgesic effect in mice with nullified expression of these receptors.

Another limitation of the current study is that BHMC was administered locally via a minimally invasive method—intra-plantar injection, which may be ideal for a proof-of-concept study in an animal model but has shown limited translational application for potential clinical use. Further formulation studies would be required to incorporate BHMC in suitable formulations for non-invasive local administration, such as gel, hydrogel, patch, etc. It is noteworthy that our previous study reported that mice that received systemic administration of BHMC, even at a high dose of 1000 mg/kg (i.p.), did not show observable adverse effects [19]. Nonetheless, skin sensitivity, allergy, and local toxicological studies would also need to be conducted to discern the pharmacological safety of BHMC for local administration. Furthermore, the distribution of BHMC after i.pl. administration should also be studied. Given that BHMC is able to exert analgesic effects via both systemic and local administration, the information on systemic circulatory distribution of this test compound via local administration would be crucial to determine the optimal dose to ensure a truly localized effect.

Lastly, we acknowledge the importance of including animals of both sexes in the experimental design, as male and female mice may show different responses to pain and analgesics. An immediate priority for the next phase of the study is to extend the study with female mice on the same model to discern if similar responses were shown in female mice.

## 5. Conclusions

In conclusion, the present study demonstrated that a novel synthetic benzilidine cyclohexanone derivative, 2,6-bis-4-(hydroxyl-3methoxybenzilidin)-cyclohexanone, possesses peripheral analgesic effects, with possible involvement of NO-independent cGMP/K_ATP_ activation and opioid receptors. Other possible mechanisms of action may include calcium and sodium channel blockage or the activation of G-protein-coupled inwardly rectifying potassium channels, but would require further experimentation to verify. Molecular docking is a potential method to investigate the interaction between the BHMC molecule and the receptors responsible for the analgesic effects of BHMC.

## Data Availability

The data can be obtained from the corresponding authors based on reasonable request.

## Abbreviations

The following abbreviations are used in this manuscript:

BHMC	2,6-bis-4-(hydroxyl-3-methoxybenzilidine)-cyclohexanone
cGMP	cyclic guanosine monophosphate
CTOP	D-Phe-Cys-Tyr-D-Trp-Orn-Thr-Pen-ThrNH2
NO	nitric oxide
nor-BNI	nor-Binaltorphimine
NOS	nitric oxide synthase
PGE_2_	prostaglandin E_2_
PKA	protein kinase A
PKC	protein kinase C
PKG	protein kinase G
